# Positive Education Interventions Prevent Depression in Chinese Adolescents

**DOI:** 10.3389/fpsyg.2019.01344

**Published:** 2019-06-12

**Authors:** Yukun Zhao, Feng Yu, Yiwen Wu, Guang Zeng, Kaiping Peng

**Affiliations:** ^1^Department of Psychology, Tsinghua University, Beijing, China; ^2^Institute of Social Psychology, Xi’an Jiaotong University, Xi’an, China; ^3^Positive Psychology Research Center, School of Social Sciences, Tsinghua University, Beijing, China

**Keywords:** positive education, intervention, depression, adolescents, China

## Abstract

Positive education aims to improve students’ academic performance as well as their well-being and character strengths. In contrast to traditional school counseling methods that are typically *post hoc* and pathological, positive education advocates a preventive and positive approach, which teaches students well-being skills that can reduce the chances of depression before it occurs. The current study tested this hypothesis by using a pseudo-experiment design. Six 8th grade classes (*N* = 173) in a Chinese school were randomly assigned into two groups. In the experiment group, students took positive education courses once a week that taught them primarily knowledge and skills related to positive emotions. Students in the control group took regular moral education courses. After one semester, the level of depression of students in the experiment group had no significant change while that of students in the control group increased significantly. The results showed that adolescent depression can be prevented by positive emotion interventions. Implications and limitations are discussed.

## Introduction

Depression is rampant among adolescents (Wickramaratne et al., 1989). The age of first onset of depression has become younger, yet many adolescents with depressive symptoms remain untreated (Weissman, 1987). Major depression is more prevalent in younger populations (Lewinsohn et al., 1993). Saluja et al. (2004) found that 18% of students in grades 6, 8, and 10 reported symptoms of depression. A report from the National Institute of Health indicated that the 1-year prevalence of depression in adolescents exceeded 4% (Thapar et al., 2012). Reports from other countries like Australia (Boyd et al., 2000), Italy (Frigerio et al., 2001), and Sweden (Olsson and Von Knorring, 1999) also found that depression was prevalent in these areas.

In China, rates of mental illness, especially depression, grew as rapid economic growth brought about dramatic social changes (Dennis, 2004). In a survey of 17,622 high school and college students from eight large Chinese cities, 44.3% reported depressive symptoms during the past week (Sun et al., 2010). Hong et al. (2009) used a Chinese version of the Children’s Depression Inventory to measure the prevalence of depression in urban junior high schools in Nanjing, China, and found that 15.7% of these adolescent students were at elevated risk of being depressed. In another study that surveyed Chinese high school students, one third reported a history of depression (Hesketh and Ding, 2005). Similar to the findings from Western countries that depression is associated with many negative psychological and behavioral outcomes (Boyd et al., 2000), studies in China also found that adolescent depression is associated with stress (Sun et al., 2010), internet addiction (Yang et al., 2014), lack of physical activity (Hong et al., 2009), smoking (Weiss et al., 2008), and suicidal ideation (Hong et al., 2016).

### A Positive Approach

Various programs are developed to treat and prevent adolescent depression, usually borrowing strategies from cognitive behavioral therapy, focusing on changing the overly negative and unrealistic thoughts of participants (Thapar et al., 2012). In a meta-analysis of depression prevention programs for children and adolescents, Stice et al. (2009) found that these preventive programs were effective in general, though the effect sizes were often small. In China, interventions using group consulting (Pei, 2006), cognitive coping (Wei, 2007), and mental health skills (Qu and Zhang, 2008) were also found to be effective.

However, the pathological approach that these therapy-based interventions adopted can have consequences. The interventions are often given to adolescents who are diagnosed with depressive symptoms or identified as having high depression risks, e.g., children of depressed parents (Garber et al., 2009). The participants may feel that they are being labeled as problematic (O’Connell et al., 2009). Meanwhile, other adolescents who may not have depressive symptoms or high risks of depression at the time can be left untreated and may become vulnerable when adversities hit them in the future.

The rise of positive psychology in recent years has shed light on a different approach. Positive psychology studies positive features of human beings like positive emotions, character strengths, engagement, and positive social institutes (Sheldon and King, 2001; Seligman and Csikszentmihalyi, 2014). It calls for scientific investigation of the positive side of human beings as much as the negative side. Seligman et al. (2005) further argued that positive psychology research could contribute to the alleviation of human suffering and mental disorders. Various positive interventions were developed and tested under rigorous experiments, using research results from positive psychology (Parks and Biswas-Diener, 2013). For example, participants randomly assigned to positive interventions like “gratitude visits” and “three good things” not only increased their happiness levels but also decreased their depressive symptoms as compared to those in a control group (Seligman et al., 2005). A meta-analysis of 51 positive interventions found that positive interventions had moderate effect sizes in both enhancing well-being and alleviating depression (Sin and Lyubomirsky, 2009).

Positive interventions are also used in positive psychotherapies to treat clients with mental disorders (Seligman et al., 2006; Rashid, 2015). In contrast to the traditional deficit-oriented approach to psychotherapy, positive psychotherapies adopt a strength-oriented approach that encourages clients to find their positive psychological resources, such as character strengths, hope, meaning of life, and social connections and prescribe them with positive activities like using their strengths, positive experiences, and prosocial behaviors. Rashid and Anjum (2008) tested a group version of positive psychotherapy in a randomized control study among middle school students. The intervention lasted 8 weeks with one 90-min session per week. The main components were Positive Introduction, Signature Strengths, Three Good Things, Savoring, and Family Strengths Tree. The level of mental health and well-being of the students in the intervention group measured by the Positive Psychotherapy Inventory (PPTI; Rashid, 2005, unpublished) were significantly higher than that in the control group after the experiment. Teachers also rated the behaviors of students in the intervention group as significantly better than those in the control group. Therefore, Rashid and Anjum (2008) argued that it is both imperative and feasible to use positive interventions to prevent mental health problems in schools.

This approach is adopted by the positive education movement, which applies positive psychology principles and interventions in education to increase both academic performance and student well-being (Seligman et al., 2009), especially through cultivating their character strengths. Waters (2011) reviewed school-based interventions used in positive education and found that they are beneficial to the psychological well-being of students. For example, the Penn Resiliency Program, which teaches students resilience skills including cognitive reframing, social skills, coping, and problem solving techniques, is proven to reduce depression in the United States, the United Kingdom, Australia, China, and Portugal (Gillham et al., 2008; Brunwasser et al., 2009; Seligman et al., 2009). Green et al. (2007) tested life coaching interventions on Australian female high school seniors and found that they reduced levels of depression significantly more than those in the wait list control group. Therefore, Seligman (2015) called on schools to implement positive education to address the high prevalence of depression.

Positive education can reduce depression because well-being is a protective factor against adolescent depression. In particular, interventions that foster positive emotions can reduce negative emotions (Fredrickson, 2001). For example, savoring, a technique that helps people to enjoy and fully engage their positive emotions, can help college students significantly reduce their depressive symptoms and negative emotions (Hurley and Kwon, 2013). Gratitude predicts low levels of depression among high school students (Froh et al., 2011), and adolescents randomly assigned to gratitude interventions reported higher life satisfaction and lower negative emotions compared to those assigned to the control group (Froh et al., 2008). Waters (2011) reviewed interventions that cultivated serenity in adolescents and found they could reduce students’ negative emotions. According to Fredrickson (2001), these interventions are effective because positive emotions broaden people’s cognitive and behavioral repertoires and build up their psychological resources. First, since positive emotions expand people’s attention and negative emotions narrow people’s attention, positive emotions can undo lingering effects on emotions by augmenting people’s attention to a broader scope (Basso et al., 1996; Niedenthal and Kitayama, 2013). This undoing effect also manifests itself in physiological mechanisms. For example, positive emotions can help people recover from cardiovascular tension elicited by negative emotions (Fredrickson et al., 2000). Second, positive emotions build psychological resilience that can help people better cope with negative emotions (Fredrickson, 2000). Therefore, positive emotions can trigger upward spirals of emotional well-being that lead to fewer negative emotions.

### Positive Education in China

The essence of positive education is concordant with traditional Chinese culture. Confucius famously said, “the gentleman is not a vessel” (*Analects*, 2:12), indicating that education should adopt a whole-person approach rather than mere accumulation of knowledge. And as pointed out by the *Inner Canon of the Yellow Emperor*, the most ancient Chinese medical classic, “The sages do not treat those who have already fallen ill, but rather those who are not yet ill” (2:7), Chinese culture emphasizes the importance of preventive programs. After the positive education movement was introduced to China (Ren, 2006), it quickly spread to many communities and schools. According to the Global Happiness Council (2018), more than 10,000 schools currently practice positive education in China.

However, though there have been many empirical studies to evaluate how positive education may enhance students’ academic performance, well-being, and character in other countries (Waters, 2011; Norrish et al., 2013; Adler, 2016), few have been done in China yet. In one of these rare attempts, Yu and Seligman (2002) found that an intervention adapted from the Penn Resiliency Program significantly reduced depressive symptoms of children randomly assigned to the intervention program than those in the control group at the post-test and the 3- and 6-month follow-ups. But in contrast to positive education programs that are typically applied to a whole-school or whole-class population, the participants in that study were at-risk children selected based on their depressive symptoms and family conflict reports. Furthermore, the intervention program in that study mainly used cognitive skills like optimistic explanatory styles (Peterson and Steen, 2002), while most positive education programs consist of components that directly contribute to human flourishing like positive emotions, interpersonal relationships, character strengths, etc. (Seligman, 2012). And since that study was done among children, we need more empirical evidence on the effectiveness of positive education programs in preventing depression in Chinese adolescents. The current study aims to fill in this research gap.

### The Current Study

In the current study, we designed a positive education program for middle school students, primarily focusing on understanding, awareness, creation, and leveraging positive emotions. All students in the experiment classes were included in the program, rather than only targeting students who were diagnosed or identified with having high risks for mental problems. Therefore, all students in the experiment classes could build better psychological resources to cope with depression prior to facing mental adversities. Furthermore, no students would have the feeling of being singled out or labeled as problematic. This approach was also positive, in which it mainly taught students how to identify and use positive emotions through positive activities, rather than focusing on correcting negative thoughts and their resulting behaviors. It was expected that these characteristics of the program would make students more willing and motivated to participate, which in turn made the program more effective.

We used a pseudo-random experiment design that randomly assigned six 8th grade classes in a Chinese school into an experiment group and a control group. The students in the experiment group took 10 weekly sessions of positive education content, and those in the control group took the usual moral education classes. We compared the levels of depression of both groups before and after the experiment to examine the effects of positive education programs on adolescent depression.

## Materials and Methods

### Participants

Participants were 8th grade students from one public middle school in the city of Chengdu, Sichuan, China. Three classes of that grade were randomly assigned into the experiment group, and three classes into the control group. A total of 173 students (81 males and 92 females, age *M* = 13.54, SD = 0.29) participated in this study, with 84 from the experiment group (37 males and 47 females) and 89 from the control group (44 males and 45 females).

### Procedure

Research for this study was approved by the Human Research Ethics Committee of Tsinghua University. Informed consent was obtained from participants. The participants were notified that all of their responses would only be accessible to the researchers.

The students completed online assessments of depression in their computer lab in the 2nd week of the semester. The 10-session positive education program began the following week. Each session took 45 min. It was delivered by the head teacher of each class. The head teachers were trained in the basics of positive psychology before the program started. We provided them with a detailed curriculum and instructions on how to run the 10 sessions and also held discussion sessions with them every other week to make sure they understood the curriculum and were delivering it correctly. We also maintained consistent communication with them throughout the entire semester, answering any questions that came up.

The students in the control group took moral education class delivered in a traditional classroom lecturing style. It consisted of three main components: moral characters like citizenship, patriotism, honesty, and filial piety; school discipline and class rules, including praising good behavior and criticizing bad behavior; and class culture building, varying from discussing slogans of class sports teams and styles of classroom decorations, to team building games.

The classes in both groups finished their curricula at the end of the semester, before the final exam. The students were asked to complete the online assessment of depression once again at this time.

### Program

As shown in Table 1, the positive education program consisted of an introductory session and three main modules: understanding emotions, fostering positive emotions, and managing negative emotions. We started the program with an introductory session on meditation because meditation can help students learn more in class while generating more positive emotions (Waters, 2011). The first major module was about the definition, feeling, expression, and science of emotions, so that the students can fully understand what emotions are and what they can do about them. Then, we taught students practical skills to foster positive emotions like gratitude and serenity. Finally, negative emotions management classes were added as we realized that the promotion of positive emotions would be more effective if the students could manage some of their most common and harmful negative emotions. We chose to teach them how to manage anxiety because it is the most prevalent negative emotion among adolescents (Kashani and Orvaschel, 1990), and anger as its consequence can be particularly severe (Feindler and Ecton, 1986; Lehnert et al., 1994). This way we limited management of negative emotions to two sessions, so that the program was more balanced, yet still focusing primarily on positive emotions.

**Table 1:** Outline of the positive education program.

Module	Session	Subject	Teaching goal
Introduction	1	Meditation	Learn how to meditate and the relationship between meditation and emotions
Understanding emotions	2	Know your emotions	Understand the definition, classifications, and combinations of emotions
	3	Express emotions	How to express your emotions and identify other people’s emotions
	4	Emotions and brains	Understand the basic neuroscience of emotions and better accept one’s emotions
Fostering positive emotions	5	Find good things	Use “three good things” to foster positive emotions
	6	Gratitude	Experience, understand, and express gratitude
	7	Savoring	Learn to savor positive emotions
	8	Serenity	Learn the benefits and methods of serenity
Managing negative emotions	9	Anxiety	Learn the physiological and psychological characteristics of anxiety and how to manage it
	10	Anger	Learn the physiological and psychological characteristics of anger and how to express anger appropriately

Walton (2014) argued that psychological interventions should be theoretically solid, context sensitive, and aim for long-lasting changes. Our intervention was designed under the framework of the Broaden-and-Build theory (Fredrickson, 2001), which predicts that fostering positive emotions can reduce negative emotions. We mainly used activities that required students’ hands-on participation to enforce habits and embodied memory, as well as practical skills that they could use in frequent daily scenarios. Both components aimed to produce long-lasting effects. And since interventions for adolescent populations should provide participants with greater respect (Yeager et al., 2018), we avoided long lectures, mandatory instructions, and quizzes, all of which could frustrate adolescents’ needs for being respected and discourage their participation. Instead, we focused on activities that generated positive emotions, and practical skills that they could use in their daily lives as they saw fit. We believe this would make the students experience more autonomy and respect during the interventions. Here is a brief description of the activities and skills in each session:

1. Meditation – Meditation by focusing on breathing can increase mindfulness (Levinson et al., 2014) and help regulate negative emotions like stress, anxiety, and depression (Goldin and Gross, 2010). Ren et al. (2011) found that after a 20 min meditation session, Chinese college students became more alert, conscious, and insightful compared to those in the control group. In this kickoff session of our intervention, we taught students basic mediation skills, like abdominal breathing and counting breaths, which will be practiced in the beginning of every following sessions to make them more mindful and engaged in the program.
2. Know your emotions – Based on Russell (2003) psychological constructs of emotion and Fredrickson (2009) classification of positive emotions, we taught students about the basic positive and negative emotions. They also learned the functions of emotions in their daily scenarios. For example, anger means they felt they might be trespassed by other students, interest means they found a field that they might gain substantial growth in.
3. Express emotions – The students discussed experiences of different emotions and learned how to express them, especially the negative emotions. They also learned the facial, verbal, and body cues that could help them identify other people’s emotions. They practiced this “emotion-reading” skill by guessing what emotions other students try to express in a charades-style game.
4. Emotions and brains – Using a model of human brains and the movie *Inside Out*, we taught students the basics of affective neuroscience (Panksepp and Watt, 2011), as well as the concept of neuroplasticity (Draganski et al., 2004). Teaching both subjects helped students gain a growth mindset (Dweck, 2006), in which emotions are manageable and controllable. They also learned the “flip your lid” technique (Siegel and Bryson, 2012) that can help them understand the source of their anger and better control this feeling.
5. Find good things – Employed the “Three Good Things” intervention in which students talked with their parents about at least three good things each day to offset the overly negative emotions brought by the natural negativity bias of humans (Baumeister et al., 2001). This intervention was proven effective in increasing happiness and decreasing depression (Seligman et al., 2005). In a team competition game, every six students were assigned into one team. They wrote down good things that happened in the past 24 h on a poster paper with colored pens, and the team with the most good things listed won.
6. Gratitude – Gratitude is one of the most important positive emotions and correlates with many positive outcomes (McCullough, 2004). Following the “three good things” exercise, students then discussed the people responsible for these good things, and their feelings toward them. The teacher summarized these feelings as gratitude and asked the students to express their gratitude on gratitude cards. Each team also chose one person or thing that they needed to thank and wrote a card to/for this person or thing.
7. Savoring – Savoring helps students to be completely submerged in the current positive emotion, so that they can take full advantage of the broaden-and-build effects of the positive emotion (Fredrickson, 2009). Students also used this technique to increase their mindfulness of the environment, especially with regard to its positive aspects (Langer, 1989). Starting from the mindful raisin-eating exercise (Kabat-Zinn, 2006), students were asked to practice savoring food during their next meal.
8. Serenity – Serenity is one of the top 10 positive emotions according to Fredrickson (2009), yet many adolescents do not feel serene in schools (Byrne et al., 2007). Students shared their experiences of serenity and discussed how to keep calm during stressful times. In an exercise, students were asked to deliver a short improvised presentation and use the serenity technique to calm themselves before the presentation.
9. Anxiety – Students learned the psychology of anxiety and how to distinguish between healthy anxiety and unhealthy anxiety (Levitt, 2015). In an improvised classroom activity that supposedly elicits anxiety, students learned how to convert unhealthy anxiety into healthy anxiety that could actually benefit their performances.
10. Anger – Students learned the physiological and psychological characteristics of anger, so that they could better identify anger within themselves and others. They shared the experiences of bad consequences caused by anger and tried to describe the feelings of anger. They also learned anger management skills and practiced them in the class.

### Measures

Depression was measured by the five items that assess symptoms of depression in the patient-reported outcome measurement information system (PROMIS) pediatric eight-item short forms (Varni et al., 2014). We used a Chinese version created in a previous study (Kern et al., 2018), which showed good reliability and validity. An example item is the statement, “In the past two weeks, I felt sad.” Participants were asked to rate their agreement with each answer choice according to a 5-point Likert scale. In this study, the Cronbach’s *α* for the depression measurement was 0.918.

We also measured and analyzed participants’ anxiety before and after the experiment. The details are presented in the Supplementary Material.

### Data Analysis

Data was analyzed with *t* tests and repeated measures ANOVA in SPSS 19.

## Results

### Random Assignment Check

An independent *t* test was conducted on the relationship between the level of depression of students in the experiment group (*M* = 1.59, SD = 0.88) and that of students in the control group (*M* = 1.69, SD = 0.86). There was no significant difference (*t* = 0.716, *p* = 0.477, SE = 0.133), indicating that the random assignment to conditions was effective.

### Effects of Intervention

**Figure 1:**
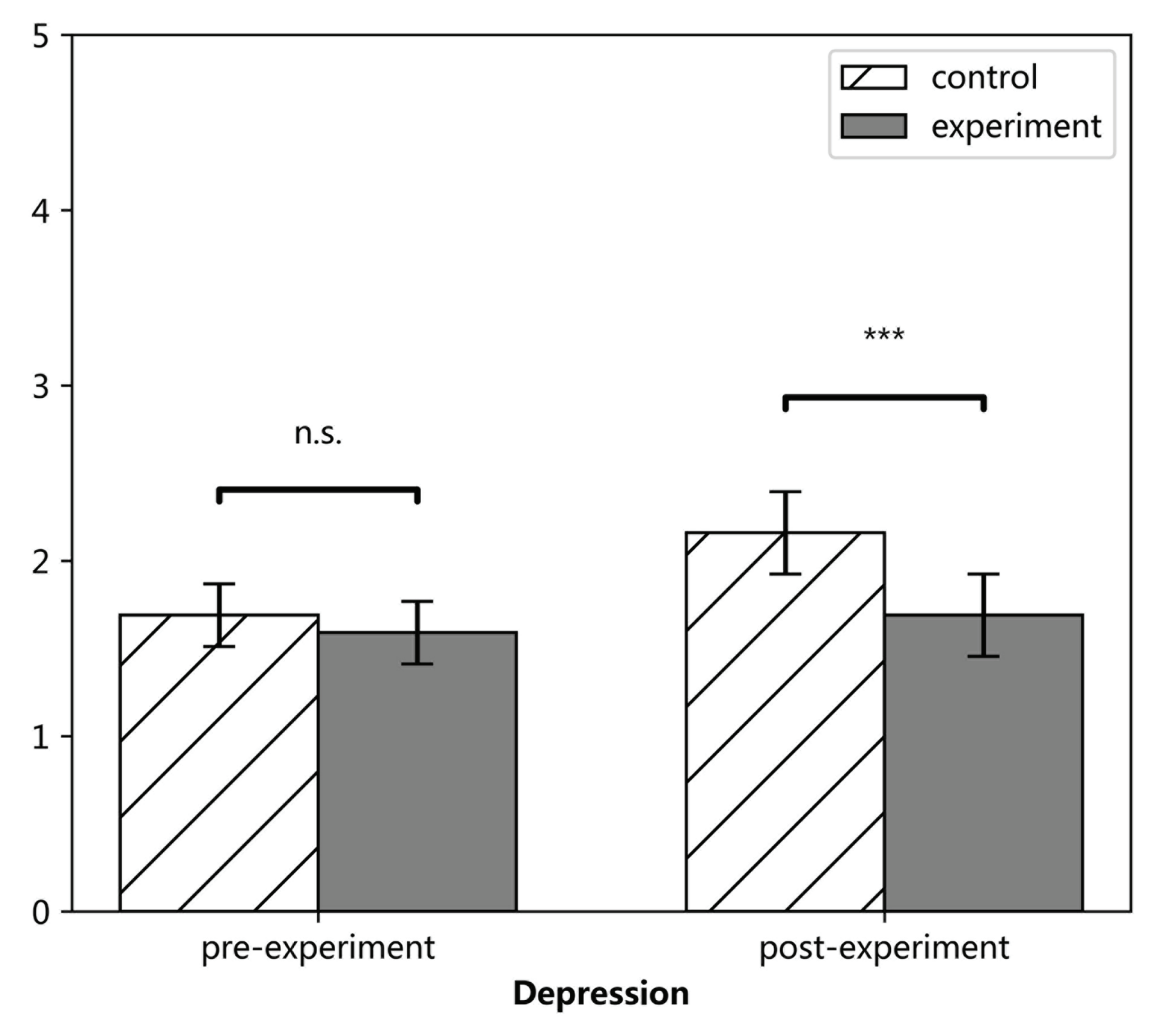
Levels of depression for groups before and after the experiment. *** *p* < 0.001.

We conducted 2 (experiment vs. control) by 2 (pre-experiment, post-experiment) ANOVA with the pre-experiment and post-experiment assessments of depression as repeated measures variables. As shown in Table 2, the main effects of both time and condition were significant. The interaction between time and assignment condition was significant too, as illustrated in Figure 1. The increase of the level of depression of students in the experiment group was significantly lesser than that in the control group. Simple effects analysis showed that there was no significant difference between the levels of depression of students in the two groups before the experiment, *F*(1, 171) = 0.509, *p* = 0.477, *η^2^* = 0.003; after the experiment, the level of depression of students in the experiment group was significantly lower than that of the control group, *F*(1, 171) = 9.691, *p* = 0.002, *η^2^* = 0.055. In the control group, the simple effect of time was significant, *F*(1, 171) = 18.218, *p* < 0.001, *η^2^* = 0.096; in the experiment group, there was no significant simple effect of time, *F*(1, 171) = 0.722, *p* = 0.397, *η^2^* = 0.004.

**Table 2:** ANOVA results of depression between experiment and control groups over time.

Effect	*F*	Hypothesis df	Error df	*p*	Partial *η^2^*
Time	12.842	1	171	<0.001	0.070
Condition	5.887	1	171	0.016	0.033
Time × condition	5.592	1	171	0.019	0.032

## Discussion

The current study attempted to test the effects of a positive education program that focused primarily on positive emotions on the prevention of depression in adolescents. The results showed that the positive education program successfully protected participating students from increases in depression.

The effect size of the current study was relatively small for two possible reasons. First, the post-experiment assessment was administered right before a final exam, when the level of negative emotions of the students were the highest during the whole semester. Even after we added anxiety and anger management sessions in the positive education program, this ceiling effect of soaring negative emotions might have cofounded the results of the current study.

Second, culture plays a role in the dynamic relationship between positive emotions and negative emotions (Mesquita and Frijda, 1992; Shweder et al., 1993). Bagozzi et al. (1999) found that the emotional styles of East Asians were more dialectical than those of Americans. Generally speaking, East Asians are more likely to experience positive emotions and negative emotions simultaneously than Euro-Americans (Schimmack et al., 2002; Spencer-Rodgers et al., 2010; Miyamoto and Ryff, 2011). Miyamoto et al. (2010) found that East Asians exhibit more negative emotions than Euro-Americans in positive situations or when asked to recall positive memories. In other words, the effects of fostering positive emotions on reducing negative emotions might be smaller for East Asians. Therefore, the dialectical emotional style of the Chinese participants in this study might have offset the effects of the positive emotion interventions on depression.

The current study is, to the best of our knowledge, the first study to test the effects of a positive education program among Chinese adolescents. Many of the psychological characteristics of Chinese students are different from their counterparts in the West. For example, they are more collectivistic (Triandis, 2001), more dialectical (Schimmack et al., 2002; Spencer-Rodgers et al., 2010; Miyamoto and Ryff, 2011), possessing more holistic thinking styles (Nisbett et al., 2001), and interdependent self-construals (Gudykunst et al., 1996). Therefore, it remained in doubt whether positive education could increase adolescent well-being in China as well. This study provided empirical evidence for the first time that positive education would be beneficial to Chinese adolescents.

This study has important implications in practice too. In contrast to pathological interventions that often focus on correcting students’ thoughts and behaviors, positive interventions consist of positive activities that can keep students intrinsically motivated. The more engaged in positive activities the students are, the more effectively the interventions can improve their lives, which would make them even more attracted to the positive education program. This upward spiral is best illustrated by an excerpt of testimony from one of the participating head teachers:

Because (positive education) requires children to communicate with their parents, and they have to talk about happy things, that brings laughter to their families, and everybody becomes happy … I am happy too. The happiest moment of mine each day is to read the ‘three good things’ of the students. The children’s writings are simple, childish, yet interesting. That kind of simple joy is contagious. I am happy, parents are happy, and children are flourishing. The whole class becomes positive and warm, students become more motivated to study. Who doesn’t like this kind of class? When you love the class, you will love learning too.

### Limitations and Future Research Directions

This study has several limitations. First, it was not a double blind study by design. The participating teachers in both the experiment and the control group knew the assignments. This might have caused observer bias by the teachers. Second, only two time points were measured for the intervention. Longer time tracking and more series of measurements are needed for future research, especially assessments that are not at the end of the semester and before the final exam. Doing so would avoid the possible ceiling effects of soaring negative emotions. Third, more indicators of adolescent mental health both positive and negative are needed to be measured in future research. This would present a more comprehensive evaluation of the effects of positive education programs on adolescents.

In the future, we hope to see more research to test the effectiveness of the intervention program in this study in other countries. In contrast to typical positive education programs that often consist of a wide range of positive psychology elements, this program was designed to focus on positive emotions. Similar studies are needed in cultures distinct from Chinese culture, so that we can better understand the interplay between culture and this positive education program. Furthermore, we found teachers participating in the positive education program enjoyed it in this study. It would be helpful to conduct empirical research to investigate the benefits of positive education to teachers’ well-being.

## Conclusion

This study found that a positive education program focusing on positive emotion interventions could prevent adolescent depression in a Chinese school. The results provided empirical evidence for the effectiveness of positive education programs in China.

## Data Availability

The datasets generated for this study are available on request to the corresponding author.

## Ethics Statement

Research for this study was approved by the Human Research Ethics Committee of Tsinghua University. Informed consent was obtained from participants. The participants were notified that all of their responses would only be accessible to the researchers.

## Author Contributions

YZ, FY, and GZ designed the study. YZ and YW collected and analyzed the data. YZ and FY wrote the manuscript. KP supervised the study and edited the final draft of the manuscript.

### Conflict of Interest Statement

The positive education interventions described in this article were designed by the authors. The authors declare that the research was conducted in the absence of any commercial or financial relationships that could be construed as a potential conflict of interest.
