# Supplementary material for: Positive Education Interventions Prevent Depression in Chinese Adolescents
Source: Front Psychol. 2019 Jun 12;10:1344. doi: 10.3389/fpsyg.2019.01344 (PMC6582777; doi:10.3389/fpsyg.2019.01344)
Supplement: Supplementary file 1 [file Table_1.doc]

# Supplementary Material

We measured anxiety using the five items that assess symptoms of Anxiety in the patient-reported outcome measurement information system (PROMIS) pediatric eight-item short forms (Varni et al., 2014). We used a Chinese version created in a previous study (Kern, Zeng, Hou, & Peng, 2018) which showed good reliability and validity. An example item is the statement, “In the past two weeks, I felt worried.” Participants were asked to rate their agreement with each answer choice according to a 5-point Likert scale. In this study, the Cronbach’s alpha for the anxiety measurement was 0.901.

An independent *t*-test was conducted on the relationship between the level of anxiety of students in the experiment group (*M* = 1.64, *SD* = 0.92) and that of students in the control group (*M* = 1.84, *SD* = 0.90) before the experiment. There was no significant difference (*t* = 0.716, *p* = 0.477).

We conducted 2 (experiment vs. control) by 2 (pre-experiment, post-experiment) ANOVA with the pre–experiment and post-experiment assessments of anxiety as repeated measures variables. As shown in Table 1, the main effect of time was significant, indicating that students became more anxious in the post-experiment assessment which took place right before the final exam. The main effect of condition was significant too, but the interaction between time and assignment condition was not significant.

Table 1. ANOVA results of anxiety between experiment and control groups over time

| Effect | F | Hypothesis *df* | Error *df* | *p* | Partial *η^2^* |
| --- | --- | --- | --- | --- | --- |
| time | 18.959 | 1 | 171 | <0.001 | 0.100 |
| condition | 7.815 | 1 | 171 | 0.006 | 0.044 |
| time * condition | 2.590 | 1 | 171 | 0.109 | 0.015 |

Simple effects analysis showed that there was no significant difference between the levels of anxiety of students in the two groups before the experiment, *F(1, 171)* = 2.141, *p* = .145, *η^2^* = 0.012; after the experiment, the level of anxiety of students in the experiment group was significantly lower than that of the control group, *F(1, 171)* = 9.343, *p* = .003, *η^2^* = 0.052, as illustrated in Fig. 1. In the control group, the simple effect of time was significant, *F(1, 171)* = 18.312, *p* < .001, *η^2^* = 0.097; in the experiment group, there was no significant simple effect of time, *F(1, 171)* = 3.661, *p* = .057, *η^2^* = 0.021.


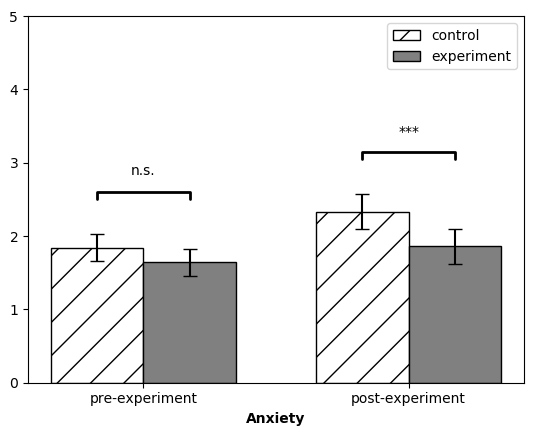


*** *p* < .001

Fig. 1 Levels of anxiety of the students before and after the experiment

# REFERENCES

Kern, M. L., Zeng, G., Hou, H., & Peng, K. (2018). The Chinese Version of the EPOCH Measure of Adolescent Well-Being: Testing Cross-Cultural Measurement Invariance. *Journal of Psychoeducational Assessment*, 0734282918789561.

Varni, J. W., Magnus, B., Stucky, B. D., Liu, Y., Quinn, H., Thissen, D., . . . DeWalt, D. A. (2014). Psychometric properties of the PROMIS® pediatric scales: precision, stability, and comparison of different scoring and administration options. *Quality of Life Research, 23*(4), 1233-1243.
